# Frequencies of emergency department use and hospitalization comparing patients with different types of substance or polysubstance-related disorders

**DOI:** 10.1186/s13011-021-00421-7

**Published:** 2021-12-18

**Authors:** Bahram Armoon, Guy Grenier, Zhirong Cao, Christophe Huỳnh, Marie-Josée Fleury

**Affiliations:** 1 Douglas Hospital Research Centre, Douglas Mental Health University Institute, 6875 LaSalle Blvd, Montreal, QC H4H 1R3 Canada; 2 Institut universitaire sur les dépendances du Centre intégré universitaire de santé et des services sociaux du Centre-Sud-de-l’Île-de-Montréal, 950 Louvain Est, Montréal, Québec H2M 2E8 Canada; 3 Department of Psychiatry, McGill University, 1033 Pine Avenue West, Montreal, QC H3A 1A1 Canada

**Keywords:** Emergency department use, Hospitalization, Substance-related disorders, Mental disorders, Clinical variables, Sociodemographic variables, Service use variables

## Abstract

**Background:**

This study measured emergency department (ED) use and hospitalization for medical reasons among patients with substance-related disorders (SRD), comparing four subgroups: cannabis-related disorders, drug-related disorders other than cannabis, alcohol-related disorders and polysubstance-related disorders, controlling for various clinical, sociodemographic and service use variables.

**Methods:**

Clinical administrative data for a cohort of 22,484 patients registered in Quebec (Canada) addiction treatment centers in 2012-13 were extracted for the years 2009-10 to 2015-16. Using negative binomial models, risks of frequent ED use and hospitalization were calculated for a 12-month period (2015-16).

**Results:**

Patients with polysubstance-related disorders used ED more frequently than other groups with SRD. They were hospitalized more frequently than patients with cannabis or other drug-related disorders, but less frequently than those with alcohol-related disorders. Patients with alcohol-related disorders used ED more frequently than those with cannabis-related disorders and underwent more hospitalizations than both patients with cannabis-related and other drug-related disorders. Co-occurring SRD-mental disorders or SRD-chronic physical illnesses, more years with SRD, being women, living in rural territories, more frequent consultations with usual general practitioner or outpatient psychiatrist, and receiving more interventions in community healthcare centers increased frequency of ED use and hospitalization, whereas both adverse outcomes decreased with high continuity of physician care. Behavioral addiction, age less than 45 years, living in more materially deprived areas, and receiving 1-3 interventions in addiction treatment centers increased risk of frequent ED use, whereas living in semi-urban areas decreased ED use. Patients 25-44 years old receiving 4+ interventions in addiction treatment centers experienced less frequent hospitalization.

**Conclusion:**

Findings showed higher risk of ED use among patients with polysubstance-related disorders, and higher hospitalization risk among patients with alcohol-related disorders, compared with patients affected by cannabis and other drug-related disorders. However, other variables contributed substantially more to the frequency of ED use and hospitalization, particularly clinical variables regarding complexity and severity of health conditions, followed by service use variables. Another important finding was that high continuity of physician care helped decrease the use of acute care services. Strategies like integrated care and outreach interventions may enhance SRD services.

**Supplementary Information:**

The online version contains supplementary material available at 10.1186/s13011-021-00421-7.

## Background

Acute care services, integrating ED use and hospitalization, are among the most expensive healthcare services [1–3]. They are well-known measures of adverse outcomes [4–6], as frequent use of acute care services is a solid indicator of poor access to services or inadequate continuity or quality of outpatient care [4, 7]. Patients with substance-related disorders (SRD), mainly alcohol [8] and cannabis [9] as the most prevalent SRD [8, 9], are more likely to use ED and be hospitalized than patients without SRD [10, 11]. Studies suggest that 27-36% of frequent ED users, defined as those making 3-4+ ED visits/year [12], have SRD [13, 14], while hospitalization rates ranged from 5 to 31% among patients with SRD for a 6-12-month period [15, 16]. Previous studies have also shown variations in acute care use for different types of SRD. Higher odds of both ED use and hospitalization were identified among patients with co-occurring opioid and cannabis-related disorders versus opioid-related disorders only [17]. Higher ED use was reported among patients with alcohol versus cocaine-related disorders [18], and among those with opioid versus alcohol or cannabis-related disorders [19]. Among patients with SRD [20], those with polysubstance-related disorders have a particularly high incidence of adverse outcomes, compared with patients affected by a single type of SRD [17, 21]. Moreover, among the polysubstance disorders, opioid-related disorders were identified as the main SRD associated with frequent hospitalizations, followed by cannabis-related-disorders [22].

Yet, few studies have compared types of SRD or polysubstance-related disorders and their respective impact on the frequency of ED use [17, 18, 19] or hospitalization [18, 22]. The SRD literature focuses mainly on single types of SRD [23]. Notwithstanding higher frequencies of ED use and hospitalization among patients with SRD, particularly polysubstance-related disorders over other types of SRD [9], other key clinical and sociodemographic variables were found to be associated with greater use of acute care services [9, 24–29]. The main associated variables included co-occurring SRD-MD [9] or SRD-chronic physical illnesses [9], being women [9, 28], low income, homelessness [18], and living in materially deprived areas [25] or rural territories [24]. Most of these studies omitted service use variables. However, previous ED visits [25, 29] and hospitalizations [24, 29] and enrollment in a health insurance program [23] were often found to increase ED use, whereas receiving more days of psychiatric care [26] and prior drug treatment [27] increased hospitalization rates.

To our knowledge, no previous study has compared both frequencies of ED use and hospitalization among patients with various types of SRD, including alcohol-related disorders, cannabis-related disorders and other drug-related disorders than cannabis, as well as polysubstance-related disorders. Outpatient service use may also protect against acute care use, especially among patients with polysubstance-related disorders. Better knowledge of the relationships among various types of SRD and other variables like physician care, psychosocial interventions and use of addiction services in terms of reducing the use of acute care services may contribute to improving overall services for these vulnerable patients. This study thus aimed to compare the frequencies of ED use and hospitalization for medical reasons among patients with four types of SRD: cannabis-related disorders, drug-related disorders other than cannabis, alcohol-related disorders, and polysubstance-related disorders, controlling for multiple clinical, sociodemographic and service use variables. We hypothesized: (1) that patients with polysubstance-related disorders would experience more frequent acute care episodes than other patients, (2) that clinical variables would be better predictors of acute care use than service use and sociodemographic variables, and (3) that higher intensity, continuity and diversity of outpatient service use would decrease ED use and hospitalization.

## Methods

### Study context

Data emanated from 14 (of 16) addiction treatment centers in Quebec (Canada). These centers are specialized regional public organizations offering SRD and behavioral addiction treatment programs like detoxification, substitution or reintegration treatments and brief intervention units. SRD services are accessible by self-referral, referral from other primary care services or by court order. They are complementary to primary care services including care provided by general practitioners (GP), over 60% of whom work in family medicine groups, or services provided by psychosocial teams (e.g., social workers, psychologists) working in community healthcare centers. Family medicine groups provide GP with additional psychosocial clinicians like nurses and social workers and enhanced secretarial support. They also insure patient registration, better access to care and care continuity through expanded days and hours of medical coverage, including walk-in clinics [30]. While some GP work in community healthcare centers on a salaried basis, most Quebec physicians are remunerated on a fee-for-service basis.

### Study design and sample

**Fig. 1:**
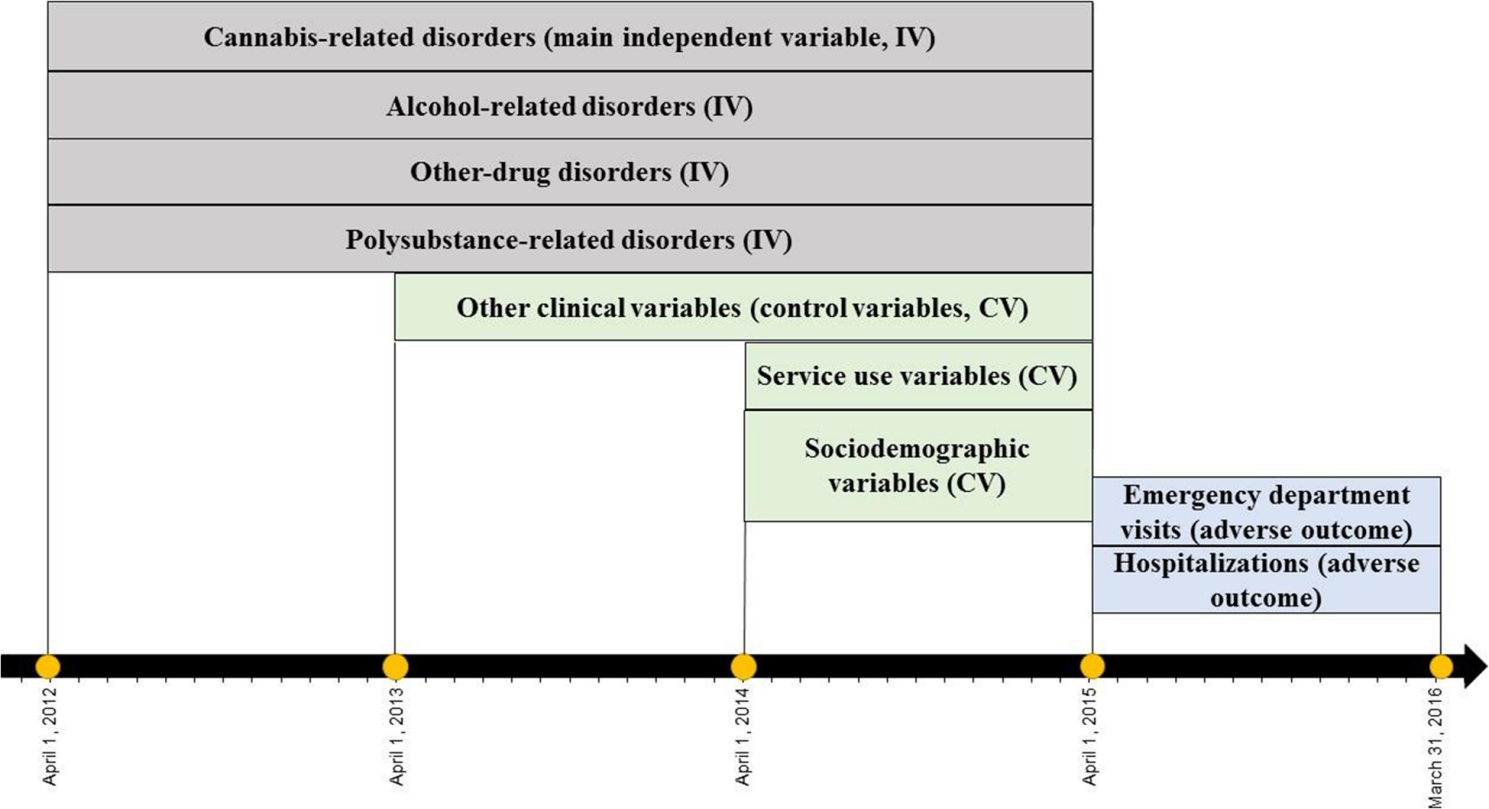
Flowchart of sample timelines and variables assessment

Data were extracted for 22,615 patients diagnosed with SRD who were registered in the 2012-13 addiction treatment center database (henceforth “SIC-SRD”). Patients had to be Quebec residents age 12+ with a RAMQ (*Régie de l’asssurance maladie du Québec*) clinical record. RAMQ, the Quebec health and social services database, integrates billing systems for most physician services, excluding 6% of services that occur outside the public system [31]. Of the 22,615 patients, those who died or were incarcerated in 2015-16, and those hospitalized in 2014-15 for more than 90 days who therefore could not be adequately assessed for outpatient care over that year were excluded. The resulting sample included 22,484 patients. The study outcomes (frequency of ED use and frequency of hospitalization) were measured in 2015-16. The main independent variables studied (specific types of SRD) were derived from 2012-13 to 2014-15 data, and included: cannabis-related disorders, drug-related disorders other than cannabis (e.g., cocaine, opioids), alcohol-related disorder and polysubstance-related disorders. The RAMQ database didn’t allow for accurate identification of other drug-related disorders than cannabis, which explains the creation of this group which also integrated drugs that usually generate very adverse outcomes [32, 33]. Control variables included other clinical, sociodemographic, and service use variables. Clinical variables were measured from 2013-14 to 2014-15, except for number of years with SRD ranging from 2009-10 to 2014-15. Sociodemographic variables were identified in 2014-15, as well as service use variables measuring care received over the 12 months prior to ED use and hospitalization (Fig. 1). The Quebec Commission for Access to Information and the ethics committee of a university health and social service organization approved the multi-site research protocol.

### Study data sources

Data from the SIC-SRD (addiction treatment center database) included patient sociodemographic and clinical characteristics, SRD, and services received for behavioral addiction. The RAMQ integrated various sub-databases from which patient information was accessed through a unique RAMQ health and social identifier matched to the SIC-SRD database. All SIC-SRD and RAMQ data from 2009-10 to 2015-16 were merged with data from the following sub-databases: the “FIPA” including individual sociodemographic and socioeconomic data, “MED-ECHO” for hospitalization data, “I-CLSC” containing data on public primary care service use in community healthcare centers, and the “BDCU” database on use of Quebec ED.

### Study variables

The two dependent variables, frequency of ED use and hospitalization, included visits for any medical reason, excluding maternity-related hospitalization. Independent clinical, sociodemographic and service use variables were identified in previous literature on SRD and acute care [9, 28, 29]. Other than the four types of SRD, clinical variables included MD, chronic physical illnesses and behavioral addictions (gambling, internet, and gaming disorders), and number of years with SRD. SRD included substance use disorders, substance intoxication, substance withdrawal and substance-induced disorders. MD encompassed common MD (e.g., anxiety, depressive, adjustment, and attention deficit/hyperactivity disorders), serious MD (bipolar, schizophrenia spectrum and other psychotic disorders) and personality disorders. The list of chronic physical illnesses was framed by the Elixhauser Comorbidity Index, identifying 31 illnesses, except for 4 conditions related to SRD or MD [34]. All diagnoses identified in RAMQ were based on the International Classification of Diseases Ninth Revision (ICD-9), and MED-ECHO and BDCU, on the Tenth Canadian Revision (ICD-10-CA) (Additional file 1).

Sociodemographic variables included age, sex, types of territory, and the material and social deprivation indices with smallest dissemination areas based on the 2011 Canadian census and determined by postal code [35]. The Material Deprivation Index integrated population employment, average income, and number of individuals without a high school diploma; while the Social Deprivation Index included numbers of individuals living alone, single individuals and single-parent families [35]. Both indices were classified in quintiles, the fifth representing highest level of deprivation. For this study, quintiles were regrouped into three levels representing the least (1-2), moderate (3) and most (4-5, not assigned) deprived areas. Patients living in areas not assigned included mainly homeless individuals and those living in nursing homes.

Service use variables included: frequency of consultations with usual GP and usual outpatient psychiatrist; high continuity of physician care; frequency of psychosocial interventions provided in community healthcare centers (excluding interventions from GP); and frequency of interventions received in addiction treatment center services. Usual GP, a proxy for patient family physician, was defined as having at least two consultations with the same GP or with at least two GP working in the same family medicine group [36]. Usual psychiatrist was defined as one that followed any patient in outpatient care at least twice. Alternatively, individuals who made only one outpatient consultation with a psychiatrist had to have consulted their GP at least twice, which was considered a proxy for collaborative care [37]. Based on the literature, highest intensity of care was defined as 4+ interventions over a 12-month follow-up period by the usual GP and usual outpatient psychiatrist, or by clinicians either at community healthcare or addiction treatment centers [38–40]. Continuity of physician care was measured with the Usual Provider Continuity Index [41], which described the proportion of visits to the usual GP and usual outpatient psychiatrist divided by total GP and outpatient psychiatrist consultations made, including consultations at walk-in clinics [41]. A score of ≥0.67 is considered high continuity of care [42].

### Data analyses

Percentages were computed for categorical variables and mean (standard deviation) or median (inter quartile range-IQR) values for continuous variables. A small intraclass correlation coefficient (ICC) (<.02) indicated that multilevel analysis was not needed. Missing values were < 1%, and complete case analysis was used [43]. Considering the distribution of the dependent variables, frequency of ED use and frequency of hospitalization, both of which were highly skewed exhibiting discrete nonnegative integers and excess zeroes, count data models were chosen. The main independent variables, the SRD subgroups (cannabis-related disorders, drug-related disorders other than cannabis, alcohol-related disorders and polysubstance-related disorders) were controlled for potentially relevant clinical, sociodemographic and service use variables, based on the SRD and service use literature [44–46]. Information criteria such as Akaike’s Information Criterion (AIC) [47] and the Bayesian Information Criterion (BIC) [48] were used for model selection, with the negative binomial (NB) regression model [49] viewed as more appropriate than the Poisson [50] and zero-inflated [51] models. Rate ratios (RRs) and 95% confidence intervals (CI) (Alpha set at 0.05) were calculated for the NB models with log link and robust standard errors [52]. Dominance analysis [53, 54] was used to determine the importance of independent variables in the models, and their relative contributions to overall fit were calculated using AIC. Sensitivity analysis was conducted to ensure robust results: the NB models were rerun, and cap values used for outcome outliers. Values greater than the cap values at the 99th percentile for frequency of ED use and frequency of hospitalization were replaced by the cap values (frequency of ED use at 20; frequency of hospitalization at 7). Statistical analyses were performed using Stata 17.

## Results

Of the 22,484 patients with SRD, 48.0% had used ED in the one-year period for which outcomes were measured, for an average 3.05 ED visits (range: 1-106; median = 2; SD = 4.18), while 17.1% of patients were hospitalized, making 1.64 hospitalizations on average (range: 1-12; median =1; SD = 1.20.). For this cohort, 9.1% (*n* = 2036) had cannabis-related disorders, 19.7% (*n* = 4420) drug-related disorders other than cannabis, 25.0% (*n* = 5627) alcohol-related disorders and 46.3% (*n* = 10,401) polysubstance-related disorders, including 2025 patients with associated cannabis and other drug-related disorders, 1511 with cannabis and alcohol-related disorders, 3957 with drug-related disorders other than cannabis and alcohol-related disorders, while 2908 had combined cannabis-related, other drug-related and alcohol-related disorders. Co-occurring SRD-MD affected 57.1% of the cohort, and SRD-chronic physical illnesses 34.3% (Table 1). Patients were 66.2% male; 44.9% ages 25-44 years, while 57.4 and 62.9% scored high on material and social deprivation (4-5, not assigned). In 2014-15, 47.5% reported no consultations with their usual GP and outpatient psychiatrist, 60.2% no intervention in community healthcare centers, while 72.1% did not use addiction rehabilitation centers, and 46.5% scored high (≥.67) on continuity of physician care. Table 2 presents clinical, sociodemographic and service use variables for the four respective types of SRD.

**Table 1:** Characteristics of patients with substance-related disorders (SRD) (*n* = 22,484)

	n/Mean	%/SD	Median	IQR
**Outcomes (2015-16)**				
Frequency of emergency department (ED) visits (Mean, SD)	1.46	3.28	0	2
Frequency of hospitalizations (Mean, SD)	0.28	0.79	0	0
**SRD: subgroups (2012-13 to 2014-15)**				
Cannabis-related disorders	2036	9.06		
Drug-related disorders other than cannabis	4,420	19.66		
Alcohol-related disorders	5,627	25.03		
Polysubstance-related disorders^a^	10,401	46.26		
**Clinical variables (2013-14 to 2014-15 or other as specified)**				
Mental disorders (MD)	12,834	57.08		
Chronic physical illnesses^b^	7,715	34.31		
Behavioral addictions (gambling, internet and gaming disorders)	360	1.60		
Number of years with SRD (2009-10 to 2015-16) (Mean, SD.)	2.74	1.69	2	3
**Sociodemographic variables (2014-15)**				
Sex				
Men	14,880	66.18		
Age				
12-17 years	831	3.70		
18-24 years	3,471	15.44		
25-44 years	10,095	44.90		
45+ years	8,087	35.97		
Material Deprivation Index				
1-2	5,739	25.52		
3	3,840	17.08		
4-5 and not assigned^c^	12,905	57.40		
Social Deprivation Index				
1 and 2	4,996	22.22		
3	3,356	14.93		
4-5 and not assigned^c^	14,132	62.85		
Types of territory:				
Urban (> 100,000)	11,789	52.48		
Semi-urban (10,000 to 100,000)	6,374	28.38		
Rural (< 10,000)	4,299	19.14		
**Service use variables (2014-15)**				
Frequency of consultations with usual general practitioner (GP) and usual outpatient psychiatrist^d^				
0-1	10,674	47.47		
2-3	4,686	20.84		
4+	7,124	31.68		
High Usual Provider Continuity Index integrating both GP and psychiatrist^d^ (≥.67)	10,446	46.46		
Frequency of interventions provided in community healthcare centers (excluding interventions from GP)				
0	13,529	60.17		
1-3	4,534	20.17		
4+	4,421	19.66		
Frequency of interventions received in addiction treatment center services^e^				
0	16,211	72.10		
1-3	1,921	8.54		
4+	4,352	19.36		

^a^ Polysubstance-related disorders group included the following sub-groups: cannabis +other drugs-related disorders (*n* = 2,025), cannabis +alcohol-related disorders (*n* = 1,511), other drugs +alcohol-related disorders (*n* = 3,957), cannabis +other drugs +alcohol-related disorders (*n* = 2,908)

^b^ Chronic physical illnesses included: renal failure, cerebrovascular illnesses, neurological illnesses, hypothyroidism, fluid electrolyte illnesses, obesity, any tumor without metastasis, metastatic cancer, chronic pulmonary illnesses, diabetes complicated and uncomplicated, congestive heart failure, peripheral vascular illnesses, valvular illnesses, myocardial infarction, hypertension, pulmonary circulation illnesses, blood loss anemia, ulcer illnesses, liver illnesses, AIDS/HIV, rheumatoid arthritis/collagen vascular illnesses, coagulopathy, weight loss, paralysis, deficiency anemia

^c^ Missing address or living in an area where index assignment is not feasible. An index cannot usually be assigned to residents of nursing home or homeless individuals

^d^ Usual GP (proxy for “patient family physician”) was defined as having at least two consultations with the same GP or with at least two GP working in the same family medicine group. Usual psychiatrist was defined as one that followed any patient in ambulatory care at least twice. Alternatively, individuals who made only one outpatient consultation with a psychiatrist had to have consulted their GP at least twice, which was considered a proxy for collaborative care. The Usual Provider Continuity Index describes the proportion of visits to the GP and psychiatrist most frequently used of all GP and psychiatrists consulted in ambulatory care

^e^ Services offered in addiction treatment centers included: medical activities (e.g. substitution treatment), specialized services for pathological gambling (e.g. rehabilitation), external services for pathological gambling (e.g. family support services), specialized addiction services (alcohol, drugs; e.g. detoxification treatment); external addiction services (e.g. reintegration), and brief treatment in addiction intervention units

**Table 2:** Patient characteristics among 4 subgroups of substance-related disorder (SRD) (n = 22,484)

	Cannabis-related disorders		Other drugs-related disorders		Alcohol-related disorders		Polysubstance-related disorders^a^	
Group size	n = 2,036 (9.06%)		n = 4,420 (19.66%)		n = 5,627 (25.03%)		n = 10,401 (46.26%)	
**Outcomes (2015-16)**	**Mean (SD)**	**Median (IQR)**	**Mean (SD)**	**Median(IQR)**	**Mean (SD)**	**Median(IQR)**	**Mean (SD)**	**Median(IQR)**
Frequency of emergency department (ED) visits	0.83 (1.59)	0 (1)	1.36 (2.48)	0 (2)	1.34 (2.94)	0 (2)	1.70 (3.91)	1 (2)
Frequency of hospitalizations	0.11 (0.40)	0 (0)	0.24 (0.68)	0 (0)	0.32 (0.86)	0 (0)	0.31 (0.84)	0 (0)
**Clinical variables (2013-14 to 2014-15 or other as specified)**								
	**n**	**%**	**n**	**%**	**n**	**%**	**n**	**%**
Mental disorders (MD)	945	46.41	2,442	55.25	3,225	57.31	6,222	59.82
Chronic physical illnesses^b^	299	14.69	1,479	33.46	2,605	46.29	3,332	32.04
Behavioral addictions (gambling, internet and gaming disorders)	15	0.74	74	1.67	96	1.71	175	1.68
Number of years with SRD (2009-10 to 2014-15) (Mean, SD)	1.66 (0.92)	1 (1)	3.09 (1.74)	3 (2)	2.63 (1.60)	2 (3)	2.86 (1.75)	2 (3)
**Sociodemographic variables (2014-15)**								
Men	1,385	68.03	2,882	65.20	3,563	63.32	7,050	67.78
Age								
12-17 years	337	16.55	52	1.18	20	0.36	422	4.06
18-24 years	791	38.85	531	12.01	215	3.82	1,934	18.59
25-44 years	718	35.27	2,455	55.54	1,826	32.45	5,096	49.00
45+ years	190	9.33	1,382	31.27	3,566	63.37	2,949	28.35
Material Deprivation Index								
1 and 2	593	29.13	1,046	23.67	1,574	27.97	2,526	24.29
3	378	18.57	758	17.15	974	17.31	1,730	16.63
4, 5 and not assigned^c^	1,065	52.31	2,616	59.19	3,079	54.72	6,145	59.08
Social Deprivation Index								
1-2	547	26.87	965	21.83	1,331	23.65	2,153	20.70
3	399	19.60	591	13.37	864	15.35	1,502	14.44
4-5 and not assigned^c^	1,090	53.54	2,864	64.80	3,432	60.99	6,746	64.86
Types of territory								
Urban (> 100,000)	898	44.17	2,503	56.68	3,049	54.27	5,339	51.36
Semi-urban (10,000 to 100,000)	733	36.06	1,093	24.75	1,471	26.18	3,077	29.60
Rural (< 10,000)	402	19.77	820	18.57	1,098	19.54	1,979	19.04
**Service use variables (2014-15)**								
Frequency of consultations with usual general practitioner (GP) and usual outpatient psychiatrist^d^								
0-1	1,201	58.99	2,016	45.61	2,461	43.74	4,996	48.03
2-3	371	18.22	916	20.72	1,358	24.13	2,041	19.62
4+	464	22.79	1,488	33.67	1,808	32.13	3,364	32.34
High Usual Provider Continuity Index integrating both GP and psychiatrist^d^ (≥.67)	742	36.44	2,105	47.62	2,900	51.54	4,699	45.18
Frequency of interventions provided in community healthcare centers (excluding interventions from GP)								
0	1,317	64.69	2,652	60.00	3,466	61.60	6,094	58.59
1-3	389	19.11	910	20.59	1,127	20.03	2,108	20.27
4+	330	16.21	858	19.41	1,034	18.38	2,199	21.14
Frequency of interventions received in addiction treatment center services^e^								
0	1,590	78.09	3,108	70.32	4,142	73.61	7,371	70.87
1-3	133	6.53	368	8.33	447	7.94	973	9.35
4+	313	15.37	944	21.36	1,038	18.45	2,057	19.78

^a^ Polysubstance-related disorders group included the following sub-groups: cannabis +other drugs-related disorders (n = 2,025), cannabis +alcohol-related disorders (n = 1,511), other drugs +alcohol-related disorders (n = 3,957), cannabis +other drugs +alcohol-related disorders (n = 2,908)

^b^ Chronic physical illnesses included: renal failure, cerebrovascular illnesses, neurological illnesses, hypothyroidism, fluid electrolyte illnesses, obesity, any tumor without metastasis, metastatic cancer, chronic pulmonary illnesses, diabetes complicated and uncomplicated, congestive heart failure, peripheral vascular illnesses, valvular illnesses, myocardial infarction, hypertension, pulmonary circulation illnesses, blood loss anemia, ulcer illnesses, liver illnesses, AIDS/HIV, rheumatoid arthritis/collagen vascular illnesses, coagulopathy, weight loss, paralysis, deficiency anemia

^c^ Missing address or living in an area where index assignment is not feasible. An index cannot usually be assigned to residents of nursing home or homeless individuals

^d^ Usual GP (proxy for “patient family physician”) was defined as having at least two consultations with the same GP or with at least two GP working in the same family medicine group. Usual psychiatrist was defined as one that followed any patient in ambulatory care at least twice. Alternatively, individuals who made only one outpatient consultation with a psychiatrist had to have consulted their GP at least twice, which was considered a proxy for collaborative care. The Usual Provider Continuity Index describes the proportion of visits to the GP and psychiatrist most frequently used of all GP and psychiatrists consulted in ambulatory care

^e^ Services offered in addiction treatment centers included: medical activities (e.g. substitution treatment), specialized services for pathological gambling (e.g. rehabilitation), external services for pathological gambling (e.g. family support services), specialized addiction services (alcohol, drugs; e.g. detoxification treatment); external addiction services (e.g. reintegration), and brief treatment in addiction intervention units

Compared to patients with cannabis-related disorders, those with alcohol or polysubstance-related disorders had a greater risk for frequent ED use and hospitalization (Table 3). Risks were 11 and 18% greater for ED use among patients with alcohol-related disorder and polysubstance-related disorders respectively, while 38 and 26% greater for hospitalization. Compared to patients with drug-related disorders other than cannabis, those with polysubstance-related-disorders had a 12% higher risk of frequent ED use, whereas patients with alcohol-related and polysubstance-related disorders had 38 and 26% higher risks of frequent hospitalization, respectively. Compared to patients with alcohol-related disorders, those with polysubstance-related disorders had a 7% higher risk of frequent ED use, but 9% less risk of frequent hospitalization (Additional file 2). Overall, the various types of SRD contributed 5.7 and 5.6% to the overall fit of both statistical models for frequency of ED use and frequency of hospitalization respectively, while the clinical, sociodemographic and service use control variables contributed 71.1, 2.5 and 20.7% respectively to the ED model, and 71.1, 4.7 and 18.6% to the hospitalization model.

**Table 3:** Negative binomial regression results on frequency of emergency department (ED) use and hospitalization among patients with substance-related disorders (SRD)

	ED use				Hospitalizations			
	RR	p value	95%CI		RR	p value	95%CI	
**SRD: subgroups (2012-13 to 2014-15)**								
Other drug-related disorders vs. cannabis-related disorders	1.06	0.253	0.96	1.17	1.00	0.998	0.83	1.20
Alcohol-related disorders vs. cannabis-related disorders	1.11	0.041	1.00	1.23	1.38	0.0001	1.15	1.65
Polysubstance-related disorders^a^ vs. cannabis-related disorders	1.18	0.0001	1.08	1.29	1.26	0.007	1.06	1.48
Alcohol-related disorders vs. other drug-related disorders	1.05	0.206	0.97	1.13	1.38	0.0001	1.23	1.54
Polysubstance-related disorders vs. other drug-related disorders	1.12	0.0001	1.05	1.19	1.26	0.0001	1.14	1.38
Polysubstance-related disorders vs. alcohol-related disorders	1.07	0.035	1.00	1.13	0.91	0.040	0.83	1.00
**Clinical variables (2013-14 to 2014-15 or other as specified)**								
Mental disorders (MD)	1.27	0.0001	1.21	1.34	1.39	0.0001	1.28	1.52
Chronic physical illnesses^b^	1.43	0.0001	1.36	1.50	1.87	0.0001	1.73	2.02
Behavioral addictions (gambling, internet and gaming disorders)	1.21	0.045	1.00	1.45	0.99	0.906	0.78	1.25
Number of years with SRD (2009-10 to 2014-15)	1.31	0.0001	1.29	1.33	1.36	0.0001	1.33	1.39
**Sociodemographic variables (2014-15)**								
Age								
12-17 vs. 45+ years	1.51	0.0001	1.34	1.71	0.77	0.046	0.59	1.00
18-24 vs. 45+ years	1.40	0.0001	1.30	1.51	1.00	0.950	0.88	1.13
25-44 vs. 45+ years	1.10	0.001	1.04	1.16	0.90	0.014	0.83	0.98
Women vs. men	1.10	0.0001	1.05	1.15	1.16	0.0001	1.08	1.25
Material Deprivation Index								
3 vs. 1-2	0.96	0.329	0.90	1.04	0.93	0.173	0.83	1.03
4-5 and not assigned^c^ vs. 1-2	1.07	0.018	1.01	1.13	1.01	0.760	0.93	1.10
Social Deprivation Index								
3 vs. 1-2	0.99	0.788	0.92	1.07	0.93	0.242	0.82	1.05
4-5 and not assigned^c^ vs. 1-2	1.01	0.734	0.95	1.07	0.99	0.785	0.89	1.09
Types of territory								
Semi-urban vs. urban	0.94	0.033	0.89	1.00	1.12	0.008	1.03	1.22
Rural vs. urban	1.16	0.0001	1.09	1.24	1.13	0.025	1.02	1.26
**Service use variables (2014-15)**								
Frequency of consultations with usual general practitioners (GP) and usual outpatient psychiatrist^d^								
2-3 vs. 0-1	1.20	0.0001	1.10	1.32	1.30	0.0001	1.13	1.49
4+ vs. 0-1	1.35	0.0001	1.23	1.49	1.57	0.0001	1.37	1.81
High Usual Provider Continuity Index integrating both GP and psychiatrist^d^ (≥.67) vs. low (<.67)	0.82	0.0001	0.76	0.89	0.83	0.003	0.74	0.94
Frequency of interventions provided in community healthcare centers (excluding interventions from GP)								
1-3 vs. 0	1.30	0.0001	1.23	1.38	1.18	0.0001	1.08	1.29
4+ vs. 0	1.48	0.0001	1.40	1.57	1.52	0.0001	1.40	1.65
Frequency of interventions received in addiction treatment center services^e^								
1-3 vs. 0	1.10	0.016	1.02	1.19	1.00	0.942	0.89	1.13
4+ vs. 0	0.98	0.595	0.93	1.04	0.91	0.030	0.83	0.99

^a^ Polysubstance-related disorders group included the following sub-groups: cannabis +other drugs-related disorders (n = 2,025), cannabis +alcohol-related disorders (n = 1,511), other drugs +alcohol-related disorders (n = 3,957), cannabis +other drugs +alcohol-related disorders (n = 2,908)

^b^ Chronic physical illnesses included: renal failure, cerebrovascular illnesses, neurological illnesses, hypothyroidism, fluid electrolyte illnesses, obesity, any tumor without metastasis, metastatic cancer, chronic pulmonary illnesses, diabetes complicated and uncomplicated, congestive heart failure, peripheral vascular illnesses, valvular illnesses, myocardial infarction, hypertension, pulmonary circulation illnesses, blood loss anemia, ulcer illnesses, liver illnesses, AIDS/HIV, rheumatoid arthritis/collagen vascular illnesses, coagulopathy, weight loss, paralysis, deficiency anemia

^c^ Missing address or living in an area where index assignment is not feasible. An index cannot usually be assigned to residents of nursing home or homeless individuals

^d^ Usual GP (proxy for “patient family physician”) was defined as having at least two consultations with the same GP or with at least two GP working in the same family medicine group. Usual psychiatrist was defined as one that followed any patient in ambulatory care at least twice. Alternatively, individuals who made only one outpatient consultation with a psychiatrist had to have consulted their GP at least twice, which was considered a proxy for collaborative care. The Usual Provider Continuity Index describes the proportion of visits to the GP and psychiatrist most frequently used of all GP and psychiatrists consulted in ambulatory care

^e^ Services offered at addiction treatment centers included: medical activities (e.g. substitution treatment), specialized services for pathological gambling (e.g. rehabilitation), external services for pathological gambling (e.g. family support services), specialized addiction services (alcohol, drugs; e.g. detoxification treatment); external addiction services (e.g. reintegration), and brief treatment in addiction intervention units

Having co-occurring SRD-MD, SRD-chronic physical illnesses and a higher number of years with SRD increased the risk of frequent ED use by 27, 43 and 31% respectively, while risk of hospitalization for the same variables was 39, 87 and 36%. Having SRD and associated behavioral addictions increased the risk of frequent ED use by 21%. Patients in the 12-17 and 25-44 age brackets were at 51 and 10% greater risk of frequent ED use, but were 23 and 10% less likely to be hospitalized, compared with patients 45 years old and over. Patients age 18-24 had a 40% higher risk of frequent ED use compared with those 45+. Women were 10% more likely to use ED and at 16% greater risk for frequent hospitalization than men. Compared with patients in urban territories, those living in rural areas were 16% more likely to use ED and at 13% higher risk for frequent hospitalization. Living in semi-urban versus urban areas decreased the risk of frequent ED use by 6%, but increased the risk of hospitalization by 12%. Compared to patients living in less materially deprived areas (1-2), those living in more deprived areas (4-5,not assigned) had a 7% higher risk of frequent ED use. Patients who made 1-3 or 4+ consultations with their usual GP and psychiatrist were 20 and 35% more likely to use ED respectively, while 30 and 57% more likely to be hospitalized. Continuity of physician care decreased the risk of higher ED use by 18%, and decreased hospitalization risk by 17%. Patients who received 1-3 or 4+ psychosocial interventions in community healthcare centers were 30 and 48% more likely to use ED, and were 18 and 52% more likely to be hospitalized. Patients with 1-3 interventions in addiction treatment centers were 10% more likely to use ED, while those with 4+ interventions were 9% less likely to be hospitalized. Finally, sensitivity analysis conducted on outlier outcomes in the final models, using the same independent variables, had a minimal effect with changes on RR smaller than 2% (Table 4).

**Table 4:** Sensitivity analysis of outliers based on outcomes of negative binomial regressions (only principal independent variables presented)

	Frequency of emergency department (ED) use			Frequency of hospitalizations		
	Original data	Cap at 20 (≤20)	Change	Original data	Cap at 7 (≤7)	Change
	RR	RR	(%)	RR	RR	(%)
**Substance-related disorder (SRD) exclusive groups (ref.: cannabis-related disorders)**						
Other drug-related disorders than cannabis	1.06	1.07	1.34	1.00	1.00	−0.16
Alcohol-related disorders	1.11	1.11	0.25	1.38	1.37	−0.70
Polysubstance-related disorders^a^	1.18	1.18	0.20	1.26	1.25	−0.06

^a^ Polysubstance-related disorders group included the following sub-groups: cannabis +other drugs-related disorders (n = 2,025), cannabis +alcohol-related disorders (n = 1,511), other drugs +alcohol-related disorders (n = 3,957), cannabis +other drugs +alcohol-related disorders (n = 2,908)

## Discussion

Nearly half of patients in this study had polysubstance-related disorders, in accordance with results of previous studies, that range from 37.8-68.5% [13, 44–46]. Patients with one SRD, like cannabis-related disorder, are at high risk of developing multiple SRD [9]. Most had MD as well, as did 47-100% of patients treated in services for SRD in a recent review [55], which confirms that co-occurring SRD-MD is the norm among patients with SRD. A third of patients were also affected by co-occurring SRD-chronic physical illnesses, as previously reported [44]. Other studies confirmed that co-occurring SRD-MD, SRD-chronic physical illnesses, and polysubstance-related disorders increase ED use and hospitalization [9, 56]. Moreover, the high rates of residency in more materially and socially deprived areas confirmed the association between SRD and both poverty [57] and loneliness [58] previously reported. Finally, during the 12-month follow-up period in the year before use of acute care was measured, most patients did not consult their GP and psychiatrist, nor did they receive care from either community healthcare or addiction treatment centers for primary or specialized outpatient care, which supports results of previous studies suggesting little use of health and social services in this population [59–61]. Yet, the overall contribution of types of SRD to the frequencies of ED use and hospitalization was modest: 5.7 and 5.6% respectively, as compared with control variables, particularly clinical (71.1%) and service use variables (18.6%). Regarding variable effect sizes, about one third of variables in the ED use model, mainly clinical and service use variables, had medium effect sizes (RR from 1.27 for MD to 1.51 for the 12-17 age group), while 40% of variables in the hospitalization model, mainly clinical variables, had medium to high effect sizes (RR from 1.30 for 2-3 consultations with GP to 1.87 for chronic physical illnesses).

The first hypothesis that patients with polysubstance-related disorders would experience more ED use and hospitalizations versus other SRD groups was confirmed for ED use but not entirely for hospitalization. Polysubstance-related disorders are associated with poorer treatment adherence and health outcomes [19]. Those patients are also reported with more deliberate self-harm [62], which may explain their more frequent use of acute care relative to other subgroups. In this study, that patients most likely to be hospitalized were those with alcohol-related disorders, and whose SRD effect size (RR 1.38) was also highest, confirmed results of a previous study evaluating early hospital readmissions [63]. Higher hospitalization rates by patients with alcohol-related disorders compared to other SRD groups may be explained by their elevated rates of co-occurring SRD-MD and/or chronic physical illnesses (e.g. cardiovascular diseases, liver diseases) [64], alcohol withdrawal [65], and their greater tendency to seek care episodes than patients with other SRD [66].

Hypothesis two was also confirmed, as clinical variables contributed more to both frequency of ED use and hospitalization among patients with SRD, for whom medium to large effect sizes also emerged on MD related to ED use (RR 1.27) and on chronic physical illnesses related to hospitalizations (RR 1.87). Those with co-occurring SRD and chronic physical illnesses are recognized as frequent ED users [25] with high hospitalization rates [67, 68]. Studies have demonstrated that most ED visits and hospitalizations among patients with SRD or MD were for physical health reasons [6], while co-occurring SRD-MD are often associated with frequent use of acute care services [69, 70]. Longer duration of SRD increased the risk of developing co-occurring chronic physical illnesses [71], another explanation for frequent ED use and hospitalization. Finally, while behavioral addiction may provoke stress-related physical health problems (e.g. hypertension, insomnia, migraine) [72, 73], frequent ED use may be more strongly associated with suicidal behaviors in this group [74], resulting from bankruptcy, unemployment, interpersonal conflicts or other psychosocial problems [72, 75].

The third hypothesis was partially confirmed, as higher continuity of physician care was the only variable contributing to decreases in both ED use and hospitalization, while higher frequency of treatment (4+ interventions) in addiction treatment centers decreased the risk of hospitalization. However, more consultations with the usual GP and interventions provided in community healthcare centers produced the largest effect sizes (RR 1.48 for 4+ interventions provided in community healthcare centers related to ED use and RR 1.57 for 4+ consultations with the usual GP related to hospitalizations). As SRD are often chronic [76, 77], continuity of care is strongly recommended for reducing the risk of relapse [76, 78]. Some studies have found that higher continuity of care played a protective role against frequent ED use among patients with MD, including SRD [79, 80], as well as hospitalization [81]. High continuity of care is a key indicator of recovery for patients with SRD, particularly those with more health problems [78]. Decreased risk of hospitalization among patients who received more intensive interventions in addiction treatment centers suggests that these specialized services offer sufficient intensity of treatment to adequately address important health issues. However, such interventions may not resolve the crisis situations that lead patients with SRD to ED. The fact that frequency of ED use and hospitalization increased despite higher numbers of consultations with usual GP and psychiatrists as well as numbers of interventions in community healthcare centers seems to indicate that these services were insufficient or inadequate to prevent ED use and hospitalization among patients with multiple and complex needs. Community healthcare centers and specialized mental health services respond to the multiple biopsychosocial needs of highly vulnerable patients [70], whereas this study found that few intensive and diversified services were available.

Regarding sociodemographic variables, patients in the 12-17 year age group showed the largest effect sizes for both ED use (RR 1.51) and hospitalizations (RR 0.77). Younger patients with SRD are known to engage in higher-risk behaviors involving alcohol or drugs [82] compared with their older counterparts, making them more vulnerable to adverse outcomes, including overdose [82, 83]. They are frequently identified as victims of stigmatization by health professionals [84], leading them to prefer self-care or informal treatment [84] and to avoid accessing addiction treatment [85]. This may explain why patients under 45 years old in this study were more likely to use ED than the 45+ group. As well, increased hospitalization among those 45+ compared with the 25-44 and 12-17 age groups may be explained by the association between chronic physical illnesses, older age and SRD [86]. The preponderant use of ED and hospitalization among women with SRD versus men has been reported previously [9, 26, 46, 87]. Women are also more vulnerable to adverse outcomes associated with SRD [46, 88, 89], which may also explain their higher risk of acute care use in this study. Patients living in rural territories, where GP and outpatient services are frequently lacking, were also more likely to use ED and to be hospitalized [24]. Surprisingly, living in semi-urban versus urban areas decreased the risk of ED use but increased hospitalization rates. It is possible that primary care services were sufficiently robust in semi-urban territories to provide a viable alternative to ED, although the ED failed to prevent hospitalization among patients with more complex health problems. Finally, living in more materially deprived or unassigned areas increased the risks of ED use and hospitalization, possibly due to their strong association with unemployment, low income, food insecurity and homelessness among patients with SRD [57].

### Limitations

First, clinical administrative databases are primarily developed for financial purposes, not research. As such, data from these sources represent only proxy measures of patient needs. Second, some key variables were not available in those databases like hospital psychosocial services, psychologist care provided in private practices or services like alcoholics anonymous. The study databases also didn’t include SRD severity, outside number of years with SRD measured on a 7-year period only. Third, drug-related disorders other than cannabis and polysubstance-related disorders included a broad range of drugs or SRD, which may have distinct impact on acute care used according to different SRD grouping. Forth, the results may not be generalizable to all patients with SRD, particularly those in healthcare systems without universal coverage or without any use of services in addiction treatment centers. Finally, one third of considered variables in the model had the medium to high effect sizes regarding to ED use, and 40% of the variables had the medium to high effect sizes regarding to hospitalization.

## Conclusions

This study was original in measuring the impact of ED use and hospitalization, comparing patients with four types of SRD including polysubstance-related disorders. The study findings showed that risk of ED use was higher among patients with polysubstance-related disorders, while patients with alcohol-related disorders had higher hospitalization rates compared with patients affected by cannabis-related and other drug-related disorders. However, variables other than types of SRD contributed substantially more to the frequencies of ED use and hospitalization, particularly other clinical variables involving complex and severe health conditions, followed by service use variables. Another important finding was that high continuity of physician care helped decrease the use of acute care services. Reinforcement of implementation of the chronic care model in primary health care services, and programs like assertive community treatment and integrated SRD-MD treatment may be indicated with a view toward improving continuity of care and decreasing acute care service use among patients with SRD, and more especially among those affected by polysubstance-related and alcohol-related disorders as well as co-occurring health and psychosocial needs. Outreach strategies may also be recommended at ED and hospital discharge for improving care, especially among younger patients and those living in more materially deprived areas.

## Supplementary Information

**Additional file 1.** Codes for substance-related disorders (SRD), mental disorders (MD) and chronic physical illnesses according to the International Classification of Diseases, Ninth or Tenth revisions.^a^

**Additional file 2 **Predicted counts for each type of substance-related disorder (SRD), controlling for all other variables in the final negative binomial regression model (*n* = 22,484).

## Data Availability

In accordance with the applicable ethics regulations for the province of Quebec, the principal investigator is responsible for keeping data confidential.

## Abbreviations

AIC: Aike’s Information Criterion
BDCU: Emergency Department Database *(Banque de données commune des urgences*)
BIC: Bayesian Information Criterion
CI: Confidence Interval
ED: Emergency Department
FIPA: Registration File of Insured Ppersons *(Fichier d’inscription des personnes assurées)*
GP: General Practitioner
I-CLSC: Community Healthcare Center Database (*Système d’information clinique et administrative des centres locaux de services communautaires*)
MD: Mental Disorder
MED-ECHO: Hospitalization Database (*Maintenance et exploitation des données pour l’étude de la clientèle hospitalière*)
NB: Negative Binomial
RR: Rate Ratio
RAMQ: Quebec Health Insurance Regime (*Régie de l’assurance maladie du Québec*)
SIC-SRD: Addiction Ttreatment Center Database (Système d’information clientèle pour les services de réadaptation dépendances)
SRD: Substance-Related Disorder
