# Supplementary material for: Frequencies of emergency department use and hospitalization comparing patients with different types of substance or polysubstance-related disorders
Source: Subst Abuse Treat Prev Policy. 2021 Dec 18;16:89. doi: 10.1186/s13011-021-00421-7 (PMC8684146; doi:10.1186/s13011-021-00421-7)
Supplement: Supplementary file 1 — Additional file 1. Codes for substance-related disorders (SRD), mental disorders (MD) and chronic physical illnesses according to the International Classification of Diseases, Ninth or Tenth revisions.a [file 13011_2021_421_MOESM1_ESM.docx]

**Additional file 1. Codes for substance-related disorders (SRD), mental disorders (MD) and chronic physical illnesses according to the International Classification of Diseases, Ninth or Tenth revisions^a^**

| **Diagnoses** | ***International Classification of Diseases, Ninth Revision (ICD-9)*** | ***International Classification of Diseases,***  ***Tenth Revision, Canada* (*ICD-10-CA)*** |
| --- | --- | --- |
| **SRD**^b^ | | |
| Cannabis-related disorder | 304.3, 305.2 (cannabis abuse or dependence) | F12.1, F12.2 (cannabis abuse or dependence);  F12.3, F12.4 (cannabis withdrawal);  F12.5-F12.9 (other cannabis-induced disorders);  F12.0, T40.7 (cannabis intoxication) |
| Alcohol-related disorders | 303.0, 303.9, 305.0 (alcohol abuse or dependence); 291.0, 291.8 (alcohol withdrawal); 291.1-291.5, 291.9, 357.5, 425.5, 535.3, 571.0-571.3 (other alcohol-induced disorders); 980.0, 980.1, 980,8, 980.9 (alcohol intoxication) | F10.1, F10.2 (alcohol abuse or dependence); F10.3, F10.4 (alcohol withdrawal); F10.5-F10-9, K70.0-K70.4, K70.9, G62.1, I42.6, K29.2, K85.2, K86.0, E24.4, G31.2, G72.1, O35.4 (other alcohol-induced disorders); F10.0, T51.0, T51.1, T51.8, T51.9 (alcohol intoxication) |
| Other drug-related disorders than cannabis | 292.0 (drug withdrawal); 292.1, 292.2, 292.8, 292.9 (drug-induced disorders); 304.0-304.2, 304.4-304.9, 305.3-305.7, 305.9 (drug abuse or dependence); 965.0, 965.8, 967.0, 967.6, 967.8, 967.9, 969.4-969.9, 970.8, 982.0, 982.8 (drug intoxication) | F11.1, F13.1, F14.1, F15.1, F16.1, F18.1, F19.1, F11.2, F13.2, F14.2, F15.2, F16.2, F18.2, F19.2 (drug abuse or dependence); F11.3-F11.4, F13.3-F13.4, F14.3-F14.4, F15.3-F15.4, F16.3-F16.4, F18.3-F18.4, F19.4-F19.4 (drug withdrawal); F11.5-F11.9, F13.5-F13.9, F14.5-F14.9, F15.5-F15.9, F16.5-F16.9, F18.5-F18.9, F19.5-F19.9 (other drug-induced disorders); F11.0, F13.0, F14.0, F15.0, F16.0, F18.0, F19.0, T40.0-T40.6, T40.8, T40.9, T42.3, T42.4, T42.6, T42.7, T43.5, T43.6, T43.8, T43.9, T50.9, T52.8, T52.9 (drug intoxication) |
| **MD** | | |
| **Common MD** | | |
| Depressive disorders | 300.4, 311.9 | F32.0- F32.3, F32.8, F32.9, F33.0- F33.3, F33.8, F33.9, F34.8, F34.9, F38.0, F38.1, F38.8, F39, F41.2 |
| Anxiety disorders | 300 (except 300.4) | F40-F48, F68 |
| Adjustment disorders | 309.0-309.4, 309.8, 309.9 | F43.0-F43.2, F43.8, F43.9 |
| Attention deficit/hyperactivity disorders | 314 | F90.0 |
| **Serious MD** | | |
| Schizophrenia spectrum and other psychotic disorders | 295, 297, 298 | F20, F21, F22, F23, F24, F25, F28, F29, F44.89 |
| Bipolar disorders | 296.0-296.6, 296.8, 296.9 | F30.0- F30.2, F30.8, F30.9, F31.0-F31.9 |
| **Personality disorders** | 301 | F60, F070, F340, F341, F488, F61 |
| **Chronic physical illnesses** |  |  |
| Renal failure | 403.0, 403.1, 403.9, 404.0, 404.1, 404.9, 585.x, 586.x, 588.0, V42.0, V45.1, V56.x | I12.0, I13.1, N18.x, N19.x, N25.0, Z49.x, Z94.0, Z99.2 |
| Cerebrovascular illnesses | 362.34, 430.x-438.x | G45.x, G46.x, I60.x-I69.x |
| Neurological illnesses | 331.9, 332.0, 332.1, 333.4, 333.5, 333.92, 334.x–335.x, 336.2, 340.x, 341.x, 345.x, 348.1, 348.3, 780.3, 784.3 | G10.x–G12.x, G13.x, G20.x, G21.x–G22.x, G25.4, G25.5, G31.8, G31.9, G32.x, G35.x, G36.x, G37.x, G40.x, G41.x, G93.1, G93.4, R47.0, R56.x |
| Endocrine illnesses (hypothyroidism, fluid electrolyte disorders and obesity) | 240.9, 243.x, 244.x, 246.1, 246.8, 278.0, 253.6, 276.x | E66.x, E00.x, E01.x, E02.x, E03.x, E89.0, E22.2, E86.x, E87.x |
| Any tumor without metastasis and metastatic cancer | 140.x-172.x, 174.x, 175.x, 179.x-195.x, 196.x–199.x, 200.x, 201.x, 202.x, 203.0, 238.6, 273.3 | C00.x–C26.x, C30.x–C34.x, C37.x–C41.x, C43.x, C45.x-C58.x, C60.x–C76.x, C77.x-C79.x, C80.x, C81.x-C85.x, C88.x, C90.0, C90.2, C96.x |
| Chronic pulmonary illnesses | 490x–505.x, 506.4, 508.1, 508.8 | I27.8, I27.9, J40.x-J47.x, J60.x-J64.x, J65.x, J66.x, J67.x, J68.4, J70.1, J70.3 |
| Diabetes complicated and uncomplicated | 250.0-250.2, 250.3, 250.4-250.9 | E10.2-E10.8, E11.2-E11.8, E13.2-E13.8, E14.2-E14.8, E10.0, E10.1, E10.9, E11.0, E11.1, E11.9, E13.0, E13.1, E13.9, E14.0, E14.1, E14.9 |
| Cardiovascular illnesses (congestive heart failure, cardiac arrhythmias, peripheral vascular illnesses, valvular illnesses, myocardial infarction, hypertension) and pulmonary circulation illnesses | 394.x–397.x, 424.x, 746.3–746.6, V42.2, V43.3, 401.x, 402.x–405.x, 437.2, 398.9, 402.0, 402.1, 402.9, 404.,404.0 404.1,404.9, 405.x ; 410.x, 412.x 415.0, 415.1, 416.x, 417.0, 417.8, 417.9, 428.x, 426.0, 426.1 426.5-426.7, 426.9, 427.0–427.4, 427.6–427.9, 437.2; 785.0, 996.01, 996.04, V45.0, V53.3, 093.x, 440.x, 441.x, 443.1– 443.9, 447.1, 557.1, 557.9, V43.4 | I05.x–I08.x, I09.1, I09.8, I10.x, I11.x–I13.x, I15.x, I67.4, I09.9, I11.0, I13.0, I13.2, I21.x, I22.x, I25.2, I25.5, I26.x, I27.x, I28.0, I28.8, I28.9, I34.x–I39.x, I42.0, 142.5I42.9, I43.x, I50.x, P29.0, I44.1–I44.3, I45.6, I45.9, I47.x–I49.x, Q23.0–Q23.3, Q23.8, Q23.9 R00.0, R00.1, , R00.8, T82.1, Z45.0, Z95.0, Z95.2, Z95.3, Z95.4, A52.0, I70.x, I71.x,I72.x, I73.0, I73.1, I73.8, I73.9, I77.1, I79.0, K55.1, K55.8, K55.9, Z95.8, Z95.9 |
| Other chronic physical illness categories (blood loss anemia, ulcer illnesses, liver illnesses, AIDS/HIV, rheumatoid arthritis/collagen vascular illnesses, coagulopathy, weight loss, paralysis, deficiency anemia) | 280.0, 280.9, 286.x, 287.1, 287.3-287.5 531.7, 531.9, 532.7, 532.9, 533.7, 533.9, 534.7, 534.9, 070.22, 070.23, 070.2; 070.3, 070.4, 070.4, 070.5, 456.0–456.2, 572.3, 572.8, 573.3, 573.4, 573.9, V42.7, 042.x–044.x, 136.1, 446.x, 701.0, 710.0–710.4, 710.5, 710.8, 710.9, 711.2, 714.x, 719.3, 720.x, 725.x, 728.5, 728.8, 260.x–263.x, 783.2, 799.4, 334.1, 342.x, 343.x, 344.0, 344.1, 344.2, 344.3, 344.4, 344.5, 344.6, 344.8, 344.9, 280.1; 280.9, 281.x, 285.9 | B20.x-B24.x, D50.0, D65–D68.x, D69.1, D69.3-D69.6 K25.7, K25.9, K26.7, K26.9, K27.7, K27.9, K28.7, K28.9, B18.x, I85.x, I86.4, I98.2, K71.1, K71.3–K71.5, K71.6, K71.7, K72.1, K72.9, K73.x, K74.x, K75.4, K76.0, K76.1, K76.3, K76.4, K76.5, K76.6, K76.8, K76.9, Z94.4, L90.0, L94.0, L94.1,L94.3, M05.x, M06.x, M08.x, M12.0, M12.3, M30.x, M31.x, M32.x–M35.x, M45.x, M46.0, M46.1, M46.8, M46.9x, G04.1, G11.4, G80.x, G81.x, G82.x, G83.x, E40.x–E46.x, R63.4, R64.x, D50.1, D50.8, D50.9, D51.x–D53.x, D63.x, D64.9 |

^a^ All diagnoses identified in MED-ECHO (*Maintenance et exploitation de données pour l’étude de la clientèle hospitalière*) prior to 2005-06, or in RAMQ for the full study period (*Régie de l’assurance maladie du Québec*) were based on the International Classification of Diseases Ninth Revision (ICD-9), which included a 4-digit code. The Canadian Tenth Revision (ICD-10-CA) was used for MED-ECHO in 2006-07+ and BDCU (*Banque de données commune des urgencies*).

^b^ SRD diagnostic codes were based on RAMQ, MED-ECHO or BDCU databases. These diagnoses were also identified in the SIC-SRD (*Système d'information clientèle pour les services de réadaptation dépendances*) based on standardized questionnaires, i.e., the adapted Quebec version of the Addiction Severity Index (IGT: *Indice de gravité d’une toxicomanie*) or the Global Appraisal of Individual Needs (GAIN). The research team had final scores only, not the raw data, for these standardized instruments administered by clinicians.
