# Supplementary material for: Frequencies of emergency department use and hospitalization comparing patients with different types of substance or polysubstance-related disorders
Source: Subst Abuse Treat Prev Policy. 2021 Dec 18;16:89. doi: 10.1186/s13011-021-00421-7 (PMC8684146; doi:10.1186/s13011-021-00421-7)
Supplement: Supplementary file 2 — Additional file 2 Predicted counts for each type of substance-related disorder (SRD), controlling for all other variables in the final negative binomial regression model (n = 22,484). [file 13011_2021_421_MOESM2_ESM.docx]

**Additional file 2. Predicted counts for each type of substance-related disorder (SRD), controlling for all other variables in the final negative binomial regression model (n=22,484)**

|  | **Emergency department (ED) use** (2015-16) | | |  | **Hospitalizations** (2015-16) | | |
| --- | --- | --- | --- | --- | --- | --- | --- |
| **SRD exclusive groups** (2012-13 to 2014-15) | **Margin** | **95%CI** | |  | **Margin** | **95%CI** | |
| Cannabis-related disorders | 1.01 | 0.93 | 1.09 |  | 0.16 | 0.13 | 0.18 |
| Drug-related disorders other than cannabis | 1.07 | 1.01 | 1.12 |  | 0.16 | 0.14 | 0.17 |
| Alcohol-related disorders | 1.12 | 1.06 | 1.18 |  | 0.21 | 0.20 | 0.23 |
| Polysubstance-related disorders**^a^** | 1.20 | 1.16 | 1.24 |  | 0.20 | 0.18 | 0.21 |

^a^ Types of SRD included in polysubstance-related disorders (n=10,401) are: (a) cannabis +other drugs-related disorders: n=2,025 patients; (b) cannabis +alcohol-related disorders: n=1,511patients; (c) other drugs than cannabis +alcohol-related disorders: n=3,957 patients; and (d) cannabis +other drugs +alcohol-related disorders: n=2,908 patients.
